# Assessment of the factor structure of the EPDS in Chinese perinatal women: a longitudinal study using multigroup confirmatory factor analysis

**DOI:** 10.3389/fpsyg.2025.1526716

**Published:** 2025-03-19

**Authors:** Huimin Guan, Bowen Sun, Li Yang, Ye Zhang

**Affiliations:** ^1^Department of Obstetrics and Gynecology, Beijing Friendship Hospital Affiliated to Capital Medical University, Beijing, China; ^2^College of Life Sciences, Beijing Normal University, Beijing, China; ^3^School of Public Health, Peking University, Beijing, China; ^4^Population Development Studies Center, Renmin University of China, Beijing, China; ^5^School of Population and Health, Renmin University of China, Beijing, China

**Keywords:** EPDS, perinatal women, factor structure, multigroup confirmatory factor analysis, reliability

## Abstract

**Introduction:**

Perinatal depression poses significant risks to the mental health of perinatal women, affecting both their well-being and their ability to care for themselves and infants. This study investigates the factor structure and reliability of the Edinburgh Postnatal Depression Scale (EPDS) across multiple time points in perinatal women in China.

**Methods:**

A total of 849 perinatal women participated in this study, with data collected at six time points: early, mid, and late pregnancy, as well as 1, 3, and 6 months postpartum. Parallel analysis and exploratory factor analysis were conducted to determine the factor structure of the EPDS. Internal consistency was assessed using Cronbach’s alpha. Multigroup confirmatory factor analysis was performed to assess measurement invariance between the antepartum (T1–T3) and postpartum (T4–T6) groups, and to assess the fit of model across the perinatal period.

**Results:**

Three-factor models fit best for the EPDS during the perinatal period when analyzed at each time points and across the perinatal period. When the same item assignment was applied to the antepartum and postpartum groups, a three-factor model for the EPDS fit well [*χ*^2^(df = 48) = 231.92, *p* < 0.001; CFI = 0.97, RMSEA = 0.06, TLI = 0.96]. The EPDS showed a Cronbach’s alpha of 0.84.

**Discussion:**

The 3-factor model of EPDS showed good reliability, internal consistency, and measurement invariance. Our findings suggested that the EPDS can effectively capture symptom variability in perinatal depression, supporting its use as a monitoring tool throughout both antepartum and postpartum.

## 1 Introduction

Over recent years, there has been a growing global focus on mental health challenges during the perinatal period. Perinatal depression serves as a crucial indicator of maternal mental well-being (Tato Fernandes et al., 2023) and is characterized by depressive symptoms that emerge during pregnancy or within the first year after childbirth (Gavin et al., 2005; Tato Fernandes et al., 2023). The worldwide prevalence of perinatal depression is estimated to be approximately 26.3% (Al-abri et al., 2023). In China, two meta-analyses reported that antepartum depression affects 19.7% of women, while postpartum depression occurs at a rate of 21.4% (Nisar et al., 2020; Liu et al., 2022; Zhang et al., 2024). EPDS is the most common traditional screening tool which is specifically designed to screen depression in antepartum and postpartum women (Park and Kim, 2022). Due to the extensive application of EPDS, its factor structure has gained increasing attention from researchers. This concern arises from the fact that the validity of the scales may be undermined if the factor structure varies significantly across different settings and patient populations.

However, there is ongoing debate regarding whether the EPDS exhibit a two-factor or three-factor model. Studies favoured a two-factor model in Canada and Peru (Rivières-Pigeon et al., 2000; Zhong et al., 2014), while other studies validated a three-factor model across English, Japanese, Spanish, and Slovak version of EPDS (Coates et al., 2017; Gutierrez-Zotes et al., 2018; Matsumura et al., 2020; Škodová et al., 2021; Lautarescu et al., 2022). In mainland of China, two studies confirmed a three-factor structure (Lau et al., 2010; Peng et al., 2024; Song et al., 2024). In addition, discrepancies in factor composition existed, with certain items excluded from factors in different cultural context. For example, items 3, 6, 7, 8, and 10 showed partial exclusions in Japanese and Hungarian studies (Kozinszky et al., 2017; Kubota et al., 2018; Matsumura et al., 2020; Saito et al., 2023).

Assessing factor structure across multiple time points was widely regarded as essential. While previous studies examined two or three time points (Matsumura et al., 2020; Škodová et al., 2021; Lautarescu et al., 2022; Saito et al., 2023), only a limited number assessed the EPDS factor structure at four or five time points (Kubota et al., 2018; Song et al., 2024). Additionally, few studies simultaneously explored the factor structure of the EPDS across both antepartum and postpartum periods, with most focusing exclusively on one period (Matsumura et al., 2020; Škodová et al., 2021; Song et al., 2024). In addition, the majority of these studies were cross-sectional studies. Therefore, this study aims to examine the factor structure, measurement invariance, and reliability of the EPDS across multiple time points using longitudinal data from perinatal women in China.

## 2 Methods

### 2.1 Study design and sample

This study was a longitudinal study of pregnant women who underwent pregnancy tests and delivered at Beijing Friendship Hospital in China. Participants were recruited from November 2019 to January 2022. Data were collected at early pregnancy within 12 weeks of the last menstrual period (T1) and at five separate time points during the perinatal period; specifically, mid-pregnancy with 22–24 weeks of gestation (T2), late pregnancy with 34–36 weeks of gestation (T3), 1 month postpartum with 2–4 weeks after delivery (T4), 3 months postpartum with 10–12 weeks after delivery (T5), and 6 months postpartum with 24–26 weeks after delivery (T6). All participants provided written informed consent after receiving both oral and written explanations of the study’s objectives and procedures. The study was approved by Ethics Committee of Beijing Friendship Hospital, Capital Medical University.

### 2.2 Data collection

Data were collected in the hospital, including information on participants’ psychiatric conditions, stress, anxiety, and depression. All participants completed the Edinburgh Postnatal Depression Scale (EPDS) questionnaires. Additional data were gathered on participants’ characteristics, including age, educational levels, employment statuses, monthly household incomes, planned pregnancy, gravidity, parity, adverse pregnancy history, and perinatal mortality experience. The results of Harman’s single-factor test suggested that common method bias was not a significant concern in this study.

### 2.3 Measures

Edinburgh Postnatal Depression Scale (EPDS) is the most frequently used self-report tools for detecting perinatal depression based on the DSM-5 criteria (Cox et al., 1987). It includes 10 items of respondents’ experience of symptoms such as laughter, enjoyment, guilt, anxiety, panic attacks, sadness, sleep disturbances, feelings of being overwhelmed, crying, and suicidal thoughts over the past 7 days (Smith-Nielsen et al., 2018). Although EPDS was originally created to evaluate depressive symptoms in postpartum women, it has also been applied to screen for antepartum depression.

Several studies have confirmed its cultural applicability, good internal consistency and adaptability in screening perinatal depression among Chinese population (Lee et al., 1998; Lau et al., 2010; Song et al., 2024). Chinese version of EPDS demonstrated high internal consistency, with a split-half reliability coefficient of 0.74 and a standardized Cronbach’s α of 0.78 (Lau et al., 2010).

### 2.4 Statistical analyses

For categorical variables, frequencies and percentage were calculated. Prior to factor analysis, specimen validity was assessed using the Kaiser-Meyer-Olkin (KMO) test and Bartletts test of sphericity to confirm the data’s suitability for factor analysis. Factor structures were examined using exploratory factor analysis (EFA) with the R packages EFA tools v0.4.4 and lavaan v0.6–17. Parallel analysis was conducted on a polychoric correlation matrix to determine the number of factors, comparing actual eigenvalues to the 95th percentile eigenvalues of 5,000 simulated random datasets. Scree plots were also consulted to determine the number of factors. EFA models with maximum likelihood extraction and oblique rotation were applied, considering factors with loadings ≥0.30 (Škodová et al., 2021; Lautarescu et al., 2022). These analyses were conducted for each of the six groups. The factor models were iteratively evaluated with two and three factors, as no existing research suggested factor structures with more than three factors for the EPDS. The best-fitting model was then subjected to multigroup confirmatory factor analysis (CFA) to assess measurement invariance across the different stages of antepartum and postpartum periods.

Model fit was evaluated using standard indices such as the root mean square error of approximation (RMSEA), the standardized root mean square residual (SRMR), the comparative fit index (CFI) and the Tucker–Lewis index (TLI). The RMSEA was considered acceptable if below 0.08, while values under 0.05 signified a good fit (Škodová et al., 2021). Similarly, an SRMR under 0.08 suggested an acceptable fit (Matsumura et al., 2020). A CFI greater than 0.90 was used to indicate an acceptable model fit, with values equal to or exceeding 0.95 representing a good fit (Škodová et al., 2021). TLI values greater than 0.90 were regarded as indicative of a well-fitting model (Škodová et al., 2021). All statistical tests were two-tailed with a significance level of α = 0.05. The semPlot v1.1.6 package was used to draw the path diagram.

Finally, the reliability of EPDS was assessed using Cronbach’s alpha (α), McDonald’s omega total (ωₜₒₜₐₗ), and McDonald’s omega hierarchical (ωₕ) coefficients. Values of α, ωₜₒₜₐₗ, and ωₕ ≥ 0.7 are considered indicative of acceptable reliability, while values ≥0.8 indicate good reliability (Revelle and Zinbarg, 2009; Revelle and Condon, 2019).

All analyses were conducted using R version 4.3.3.

## 3 Results

### 3.1 Sample characteristics

The study enrolled a total of 905 participants. Among them, 56 individuals were excluded due to missing sociodemographic information. Therefore, the final sample comprised 849 individuals, all of whom were surveyed in the early stages of pregnancy. The sociodemographic and reproductive characteristics of the study population are detailed in Table 1.

**Table 1:** Sociodemographic and reproductive characteristics of the sample.

	Antepartum sample			Postpartum sample		
	T1	T2	T3	T4	T5	T6
Total	849	581	450	259	151	49
Age						
18–29	277 (32.6%)	184 (31.7%)	139 (30.9%)	96 (37.1%)	45 (29.8%)	22 (44.9%)
30–35	436 (51.4%)	299 (51.5%)	238 (52.9%)	123 (47.5%)	80 (53%)	19 (38.8%)
>35	136 (16%)	98 (16.9%)	73 (16.2%)	40 (15.4%)	26 (17.2%)	8 (16.3%)
Educational level						
High school and below	61 (7.2%)	41 (7.1%)	32 (7.1%)	15 (5.8%)	7 (4.6%)	3 (6.1%)
College or above	788 (92.8%)	540 (92.9%)	418 (92.9%)	244 (94.2%)	144 (95.4%)	46 (93.9%)
Employment status						
Civil Servants and Management Personnel of Enterprises and Institutions	119 (14.1%)	78 (13.1%)	69 (14.4%)	40 (15.5%)	25 (16.9%)	10 (20.4%)
General Clerks/General Staff	381 (44.9%)	268 (46.1%)	199 (44.2%)	119 (45.9%)	70 (46.4%)	18 (36.7%)
Professional and Technical Personnel or Service Industry	145 (17.4%)	101 (17.3%)	102 (22.9%)	54 (21.1%)	25 (16.9%)	7 (14.6%)
Others	194 (23.5%)	134 (22.9%)	104 (23.3%)	54 (21.1%)	31 (20.5%)	12 (24.5%)
Monthly household income (CNY)						
0–5,000	72 (8.5%)	47 (8%)	37 (8.2%)	23 (8.8%)	10 (6.6%)	5 (10.2%)
5,001–10,000	217 (25.6%)	151 (26%)	107 (23.8%)	67 (25.9%)	41 (27.2%)	15 (30.6%)
10,001–20,000	288 (33.9%)	195 (33.6%)	162 (36%)	88 (34%)	57 (37.7%)	15 (30.6%)
>20,000	272 (32%)	188 (32.4%)	144 (32%)	81 (31.3%)	43 (28.5%)	14 (28.6%)
Planned pregnancy						
Yes	678 (79.9%)	485 (83.5%)	376 (83.6%)	205 (79.2%)	128 (84.8%)	41 (83.7%)
No	171 (20.1%)	96 (16.5%)	74 (16.4%)	54 (20.8%)	23 (15.2%)	8 (16.3%)
Gravidity(including the current one)						
1	414 (48.8%)	289 (49.7%)	230 (51.1%)	141 (54.4%)	68 (45%)	29 (59.2%)
2	297 (35%)	202 (34.8%)	151 (33.6%)	76 (29.3%)	55 (36.4%)	11 (22.4%)
3 or more	138 (16.3%)	90 (15.5%)	69 (15.3%)	42 (16.2%)	28 (18.5%)	9 (18.4%)
Parity(excluding the current one)						
0	562 (66.2%)	386 (66.4%)	301 (66.9%)	173 (66.8%)	98 (64.9%)	35 (71.4%)
1	263 (31%)	181 (31.2%)	137 (30.4%)	81 (31.3%)	50 (33.1%)	14 (28.6%)
More than 1	24 (2.8%)	14 (2.4%)	12 (2.7%)	5 (1.9%)	3 (2%)	0
Adverse pregnancy history						
No	550 (64.8%)	379 (65.2%)	298 (66.2%)	172 (66.4%)	96 (63.6%)	35 (71.4%)
Yes	299 (35.2%)	202 (34.8%)	162 (33.8%)	87 (33.6%)	55 (36.4%)	14 (38.6%)
Perinatal mortality experience						
No	23 (2.7%)	18 (3.1%)	12 (2.7%)	8 (3.1%)	4 (2.6%)	0
Yes	826 (97.3%)	563 (96.9%)	438 (97.3%)	251 (96.9%)	147 (97.4%)	49 (100%)

T1 represents the early pregnancy group, T2 represents the mid-pregnancy group, T3 represents the late pregnancy group, T4 represents the 1 month postpartum group, T5 represents the 3 months postpartum group, and T6 represents the 6 months postpartum group. CNY refers to Chinese Yuan Renminbi, which is the official currency of the People’s Republic of China.

### 3.2 Parallel analysis

The number of factors identified by the parallel analyses with principal component analysis (PCA), exploratory factor analysis (EFA) and squared multiple correlation (SMC) for the EPDS across different time points were presented in Table 2. Based on the results of parallel analysis and the scree plot, combined with existing research recommendations on factor structures, we adopted a 2- and 3-factor modeling approach.

**Table 2:** Parallel analysis of EPDS.

	T1	T2	T3	T4	T5	T6
PCA	2	1	1	1	1	1
EFA	3	3	3	2	1	1
SMC	3	4	4	3	2	3

T1 represents the early pregnancy group, T2 represents the mid-pregnancy group, T3 represents the late pregnancy group, T4 represents the 1 month postpartum group, T5 represents the 3 months postpartum group, and T6 represents the 6 months postpartum group. PCA = principal component analysis; EFA = exploratory factor analysis; SMC = squared multiple correlation.

### 3.3 Exploratory factor analysis

Exploratory factor analyses (EFAs) were conducted for the EPDS to compare the two-factor and three-factor models suggested by parallel analysis at six time points (Table 3). Table 3 displayed the factor loadings obtained from various factor extraction and rotation techniques for each item of the EPDS scale. Bolded values in the table indicate the highest loadings for each item on its corresponding factor, signifying the strongest associations. For example, in the two-factor model at T1, items 1–2, items 7–9 were loaded on F1, and items 3–6 were loaded on F2. Item 10 was not retained in the antepartum factor analyses as it did not meet the criterion of >0.30 for significant factor loading. However, in the postpartum analyses, item 10 was loaded, indicating its relevance in the postpartum period.

**Table 3:** Item-level exploratory factor analysis of the EPDS.

	2-factors models											
	Antepartum sample						Postpartum sample					
	T1		T2		T3		T4		T5		T6	
	F1	F2	F1	F2	F1	F2	F1	F2	F1	F2	F1	F2
Item 1	**−0.48**	0.06	0.12	**1.06**	**−0.57**	0.14	−0.17	**0.62**	**−0.46**	−0.16	−0.04	**−0.60**
Item 2	**−0.44**	−0.02	−0.23	**0.46**	**−0.56**	0.07	0.05	**0.99**	**−0.87**	0.13	**−0.54**	−0.03
Item 3	−0.09	**0.57**	**0.53**	0.04	0.04	**0.77**	**0.79**	0.13	0.36	**0.36**	**0.69**	0.09
Item 4	0.16	**0.65**	**0.64**	−0.04	**0.52**	0.30	0.80	0.05	**0.59**	0.27	**0.68**	0.05
Item 5	0.27	**0.45**	**0.73**	0.10	**0.47**	0.23	**0.69**	−0.15	0.04	**0.97**	**0.50**	0.03
Item 6	0.06	**0.58**	**0.65**	−0.03	**0.47**	0.29	**0.67**	−0.13	**0.62**	0.03	−0.18	**0.93**
Item 7	**0.40**	0.21	**0.64**	0.00	**0.53**	0.12	**0.78**	−0.01	**0.70**	0.03	**0.92**	−0.16
Item 8	**0.86**	−0.03	**0.67**	−0.10	**0.75**	0.03	**0.68**	−0.13	**0.86**	−0.01	0.17	**0.69**
Item 9	**0.67**	−0.01	**0.54**	−0.14	**0.64**	−0.05	**0.70**	−0.04	**0.79**	0.11	0.07	**0.36**
Item 10	0.15	0.05	0.11	−0.18	0.05	0.03	**0.62**	0.03	**0.64**	−0.08	**0.54**	0.02

	2-factors models											
	Antepartum sample						Postpartum sample					
	T1		T2		T3		T4		T5		T6	
	F1	F2	F1	F2	F1	F2	F1	F2	F1	F2	F1	F2
Item 1	**−0.48**	0.06	0.12	**1.06**	**−0.57**	0.14	−0.17	**0.62**	**−0.46**	−0.16	−0.04	**−0.60**
Item 2	**−0.44**	−0.02	−0.23	**0.46**	**−0.56**	0.07	0.05	**0.99**	**−0.87**	0.13	**−0.54**	−0.03
Item 3	−0.09	**0.57**	**0.53**	0.04	0.04	**0.77**	**0.79**	0.13	0.36	**0.36**	**0.69**	0.09
Item 4	0.16	**0.65**	**0.64**	−0.04	**0.52**	0.30	0.80	0.05	**0.59**	0.27	**0.68**	0.05
Item 5	0.27	**0.45**	**0.73**	0.10	**0.47**	0.23	**0.69**	−0.15	0.04	**0.97**	**0.50**	0.03
Item 6	0.06	**0.58**	**0.65**	−0.03	**0.47**	0.29	**0.67**	−0.13	**0.62**	0.03	−0.18	**0.93**
Item 7	**0.40**	0.21	**0.64**	0.00	**0.53**	0.12	**0.78**	−0.01	**0.70**	0.03	**0.92**	−0.16
Item 8	**0.86**	−0.03	**0.67**	−0.10	**0.75**	0.03	**0.68**	−0.13	**0.86**	−0.01	0.17	**0.69**
Item 9	**0.67**	−0.01	**0.54**	−0.14	**0.64**	−0.05	**0.70**	−0.04	**0.79**	0.11	0.07	**0.36**
Item 10	0.15	0.05	0.11	−0.18	0.05	0.03	**0.62**	0.03	**0.64**	−0.08	**0.54**	0.02

	3-factors models																	
	Antepartum sample									Postpartum sample								
	T1			T2			T3			T4			T5			T6		
	F1	F2	F3	F1	F2	F3	F1	F2	F3	F1	F2	F3	F1	F2	F3	F1	F2	F3
Item 1	0.03	0.07	**0.72**	0.05	**1.01**	−0.07	0.00	−0.07	**−0.62**	−0.12	**0.80**	0.08	−0.04	**0.44**	−0.21	**−0.56**	0.03	−0.20
Item 2	−0.04	−0.08	**0.47**	−0.16	**0.44**	−0.17	−0.11	−0.11	**−0.46**	0.08	**0.82**	−0.13	−0.05	**0.95**	0.09	0.01	−0.06	**−0.73**
Item 3	**0.57**	−0.05	0.07	−0.04	−0.07	**0.62**	**0.76**	−0.08	−0.06	**0.74**	0.08	0.04	**0.39**	0.02	0.37	0.13	**0.71**	0.02
Item 4	**0.69**	0.04	−0.10	0.27	−0.08	**0.45**	**0.56**	−0.01	0.38	**0.83**	0.00	−0.03	0.21	**−0.40**	0.31	0.08	**0.67**	0.05
Item 5	**0.52**	0.11	−0.12	**0.42**	0.08	0.38	**0.39**	0.20	0.15	**0.63**	−0.25	0.01	−0.01	−0.04	**0.99**	−0.01	0.09	**0.61**
Item 6	**0.59**	0.04	0.00	0.13	−0.11	**0.60**	**0.48**	0.13	0.21	**0.53**	−0.14	0.16	**0.52**	−0.09	0.07	**0.93**	0.04	−0.17
Item 7	0.27	0.14	−0.27	**0.48**	0.02	0.25	0.24	0.25	0.26	**0.72**	−0.04	0.06	**0.36**	−0.35	0.08	−0.13	**0.83**	0.16
Item 8	0.13	**0.48**	−0.32	**0.74**	0.00	0.11	−0.08	**1.02**	0.06	−0.04	−0.05	**0.91**	**1.03**	0.10	−0.04	**0.66**	0.27	−0.03
Item 9	−0.09	**0.90**	0.02	**0.61**	−0.05	0.07	0.07	**0.40**	0.26	0.24	−0.04	**0.53**	**0.60**	−0.19	0.15	**0.32**	−0.09	0.28
Item 10	0.04	0.20	0.01	0.28	−0.12	−0.11	0.06	0.05	−0.05	0.29	0.00	**0.36**	**0.54**	−0.10	−0.05	−0.01	0.15	**0.57**

T1 represents the early pregnancy group, T2 represents the mid-pregnancy group, T3 represents the late pregnancy group, T4 represents the 1 month postpartum group, T5 represents the 3 months postpartum group, and T6 represents the 6 months postpartum group. Bold fonts indicate items scale assignments.

### 3.4 Multigroup confirmatory factor analysis

Table 4 presents CFA fit indices for the 2- and 3-factor models of the EPDS across the six specific groups. The results indicated a consistent superior fit for the three-factor model compared to the two-factor model at each time point, except that the two-factor was slightly better at T5. For instance, at T1, the three-factor model demonstrated a CFI of 0.98 and a RMSEA of 0.05, outperforming the two-factor model which had a CFI of 0.95 and an RMSEA of 0.07. Longitudinal comparison of the fit of the three-factor model at different time points showed that the three-factor model was always well fitted. CFI was always above 0.95 and RMSEA was always less than 0.08.

**Table 4:** Confirmatory factor analysis indices of the two-factor and three-factor models of the EPDS.

	Antepartum sample						Postpartum sample					
	T1		T2		T3		T4		T5		T6	
	2FM	3FM	2FM	3FM	2FM	3FM	2FM	3FM	2FM	3FM	2FM	3FM
*χ*^2^ value	96.77	53.83	101.25	80.01	115.48	49.54	65.04	57.90	58.49	58.78	37.24	25.67
df	19	17	26	24	27	17	26	32	27	25	34	32
RMSEA	0.07	0.05	0.07	0.06	0.09	0.07	0.08	0.06	0.09	0.10	0.04	0.00
CFI	0.95	0.98	0.96	0.97	0.92	0.97	0.97	0.98	0.96	0.95	0.98	1.00
SRMR	0.04	0.03	0.04	0.04	0.05	0.04	0.04	0.04	0.04	0.05	0.08	0.06

T1 represents the early pregnancy group, T2 represents the mid-pregnancy group, T3 represents the late pregnancy group, T4 represents the 1 month postpartum group, T5 represents the 3 months postpartum group, and T6 represents the 6 months postpartum group. 2FM for the two-factor model and 3FM for the three-factor model. df for degrees of freedom, RMSEA for root mean square error of approximation, CFI for comparative fit index, and SRMR for standardized root mean square residual.

**Figure 1:**
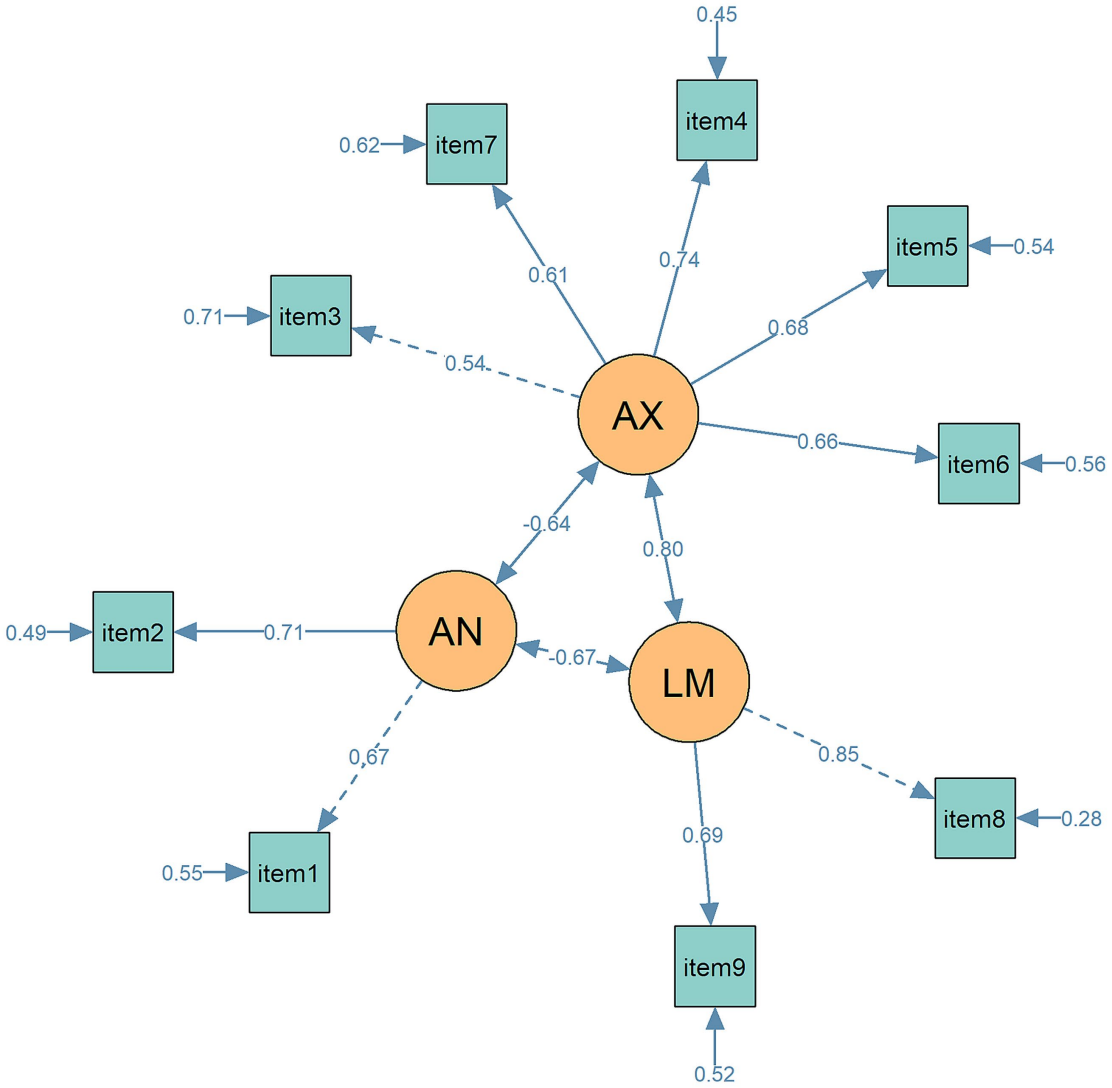
The path diagram for the 3-factor model of EPDS in CFA. Circles represent latent variables (AN = anhedonia, AX = anxiety, LM = low mood); rectangles represent observed variables (items). Standardized factor loadings and path coefficients are displayed next to each path. Lines represent measurement paths from latent variables to their respective indicators. Solid lines indicate statistically significant paths (*p* < 0 0.05), while dashed lines indicate non-significant paths. Numbers adjacent to single-headed arrows represent standardized path coefficients, and numbers adjacent to the small arrows pointing to observed variables indicate residual variances.

The EFAs identified three-factor models for the EPDS, however, factor structure varied slightly at the six time points. Therefore, we grouped T1-T3 as the antepartum period and T4-T6 as the postpartum period to test measurement invariance. We obtained a three-factor model: (1) anhedonia (items 1 and 2), (2) anxiety (items 3,4,5,6, and 7), (3) low mood (items 8 and 9). For the antepartum group, the three-factor model demonstrated good fit, with statistical indices as follows: *χ*^2^ (df = 24) = 179.23, *p* < 0.001; CFI = 0.97, RMSEA = 0.06, TLI = 0.95. For the postpartum group, the model fit was enhanced: *χ*^2^ (df = 24) = 169.326, *p* = 0.001; CFI = 0.99, RMSEA = 0.05, TLI = 0.98. The path diagram illustrating the three-factor model was presented in Figure 1.

Table 5 presents the fit indices for the multigroup confirmatory factor analysis models of the data as the antepartum and postpartum groups, each imposing stricter equality constraints. The table includes chi-square difference tests to compare the fit of each subsequent model with the preceding one. The baseline model of the EPDS showed good model fit [*χ*^2^(48) = 231.92, CFI = 0.97, RMSEA = 0.06, SRMR = 0.03]. Equating the factor loadings across groups did not significantly affect the model fit (*p* = 0.25). However, when item thresholds were also constrained to be equal, the model fit deteriorated significantly according to the chi-square test (p < 0.001), although the fit indices remained within an acceptable range (CFI = 0.97, RMSEA = 0.06, SRMR = 0.04). It provided support for measurement invariance across groups based on the three-factor model of the EPDS.

**Table 5:** Fit statistics and likelihood ratio tests of equality constraints across perinatal groups for three-factor model of EPDS.

Equality constraints	CFI	TLI	RMSEA	SRMR	*χ* ^2^	df	*χ*^2^ diff.	df diff.	p
None	0.97	0.96	0.06	0.03	231.92	48			
Loadings	0.97	0.96	0.05	0.03	239.73	54	7.809	6	0.25
Loadings and thresholds	0.97	0.96	0.06	0.04	282.48	60	42.758	6	< 0.001

CFI for comparative fit index, TLI for Tucker-Lewis index, RMSEA for root mean square error of approximation, SRMR for standardized root mean square residual, df for degrees of freedom, *χ*^2^ diff. for Chi-square difference, and df diff. for degrees of freedom difference.

### 3.5 Reliability

As shown in Table 6, the EPDS presented a Cronbach’s alpha of 0.84 and an Omega Total of 0.86, supporting adequate internal consistency and strong overall reliability by accounting for both common and specific factors influencing item responses. The Omega Hierarchical was 0.73, indicating a substantial degree of variance attributable to the general factor in the hierarchical three-factor model. The average inter-item correlation for the EPDS was 0.13, suggesting a relatively low association between individual items, which reflected the diversity in the items’ contribution to the overall scale score.

**Table 6:** Reliability statistics for EPDS scores.

Scale	Cronbach’s alpha	Omega total	Omega hierarchical	Average inter-item correlation
EPDS	0.84	0.86	0.73	0.13

## 4 Discussion

This study indicated that the three-factor models of the EPDS exhibited a better fit than two-factor models for perinatal woman in China. The EPDS demonstrated good measurement invariance, internal consistency, and reliability among Chinese perinatal woman.

Our study confirmed previous findings in favor of the three-factor model of EPDS (Coates et al., 2017; Matsumura et al., 2020; Škodová et al., 2021; Lautarescu et al., 2022; Saito et al., 2023; Peng et al., 2024; Song et al., 2024). This finding indicated that the EPDS is not only culturally adaptable but also maintains its psychometric integrity across different linguistic and cultural settings. This international consensus on the EPDS’s structure and reliability underscores its importance as a screening tool for perinatal depression (Srisurapanont et al., 2023; Stefana et al., 2023; Liu et al., 2024). Although the two-factor model performed slightly better than three-factor model at 3 months postpartum, the three-factor model still showed a good fit, with indices exceeding 0.95. The reduced sample size at this time point may also contribute to structural instability.

The item assignment in our study was not completely consistent with previous studies (Coates et al., 2017; Matsumura et al., 2020; Škodová et al., 2021; Lautarescu et al., 2022; Saito et al., 2023; Peng et al., 2024; Song et al., 2024). The differences may be attributed to cultural differences in language expression of depressive symptoms. Item 10 did not load onto any factors during the antepartum period, however, it exhibited significant loading during the postpartum period. Several reasons may explain this discrepancy. First, item 10 uniquely addresses self-harm. This may reflect the psychological focus of women can shift dramatically following childbirth. The notable loading of item 10 in the postpartum period suggests that depressive symptoms may manifest more acutely after delivery (Pope et al., 2013). Second, cultural and contextual factors may also contribute to the differing factor loadings. Societal expectations surrounding motherhood, the physical and emotional adjustments that occur post-delivery, and cultural stigmas related to mental health may influence how depressive symptoms are expressed during these distinct periods (Roomruangwong and Epperson, 2011; Batt et al., 2020). This finding also confirms that, as its name suggests, the EPDS was originally designed to detect postpartum depression.

Another study involving Chinese perinatal women using item response theory methods identified similar deficiencies in item 10. However, in contrast to their findings, item 3 demonstrated satisfactory performance in our analysis, indicating a need for further research (Peng et al., 2024).

The fit indices from our analysis substantiated the efficacy of the EPDS as an assessment tool specifically adapted for detecting perinatal depression, including anxiety symptoms (Cox et al., 1987). Our reliability metrics confirmed the robustness of the EPDS in evaluating depressive symptoms among perinatal women, aligning with results from other studies in the perinatal field (Lee et al., 1998; Lau et al., 2010; Park and Kim, 2022; Song et al., 2024).

The strengths of this study include the detailed examination of the factor structures of the EPDS across six time points, spanning the antepartum to postpartum periods, using a longitudinal sample. As far as we know, this is the study to include the largest time points to date. In addition, the 3-factor model of the EPDS were compared between the antepartum and postpartum groups, thus their reliability and internal consistency were further thoroughly tested.

There were limitations also need to be mentioned. First, the sample came from one hospital in Beijing which may restrict the generalizability of the findings to other regions of mainland of China, particularly underdeveloped areas. Second, the data may be underrepresented due to the small sample size at some time points in postpartum period.

## 5 Conclusion

This study indicates that the three-factor structure of the EPDS provides a better fit than the two-factor structure across multiple time points in Chinese perinatal women. The EPDS demonstrated good internal consistency and measurement invariance, supporting its reliability for tracking symptom changes throughout the perinatal period. However, the wording of item 10 needs refinement to enhance its applicability in the antepartum period within the Chinese context. Future research should focus on testing the scale in a larger postpartum sample and further refining item clarity to improve its cultural and temporal applicability.

## Data Availability

The raw data supporting the conclusions of this article will be made available by the authors, without undue reservation.
